# Co-Expression Network Analysis of MicroRNAs and Proteins in Severe Traumatic Brain Injury: A Systematic Review

**DOI:** 10.3390/cells10092425

**Published:** 2021-09-14

**Authors:** Claire Osgood, Zubair Ahmed, Valentina Di Pietro

**Affiliations:** 1 Neuroscience and Ophthalmology Group, Institute of Inflammation and Ageing, University of Birmingham, Edgbaston, Birmingham B15 2TT, UK; CXO738@student.bham.ac.uk; 2 Centre for Trauma Sciences Research, University of Birmingham, Edgbaston, Birmingham B15 2TT, UK; 3 Surgical Reconstruction and Microbiology Research Centre, National Institute for Health Research, Queen Elizabeth Hospital, Birmingham B15 2TH, UK

**Keywords:** traumatic brain injury, microRNAs, proteins, target discovery, bioinformatics

## Abstract

Traumatic brain injury (TBI) represents one of the leading causes of mortality and morbidity worldwide, placing an enormous socioeconomic burden on healthcare services and communities around the world. Survivors of TBI can experience complications ranging from temporary neurological and psychosocial problems to long-term, severe disability and neurodegenerative disease. The current lack of therapeutic agents able to mitigate the effects of secondary brain injury highlights the urgent need for novel target discovery. This study comprises two independent systematic reviews, investigating both microRNA (miRNA) and proteomic expression in rat models of severe TBI (sTBI). The results were combined to perform integrated miRNA-protein co-expression analyses with the aim of uncovering the potential roles of miRNAs in sTBI and to ultimately identify new targets for therapy. Thirty-four studies were included in total. Bioinformatic analysis was performed to identify any miRNA–protein associations. Endocytosis and TNF signalling pathways were highlighted as common pathways involving both miRNAs and proteins found to be differentially expressed in rat brain tissue following sTBI, suggesting efforts to find novel therapeutic targets that should be focused here. Further high-quality investigations are required to ascertain the involvement of these pathways and their miRNAs in the pathogenesis of TBI and other CNS diseases and to therefore uncover those targets with the greatest therapeutic potential.

## 1. Introduction

Traumatic brain injury (TBI) has been described as a problem of epidemic magnitude, with an estimated annual incidence of over 10 million and rising [1,2,3,4,5,6]. As such, TBI represents one of the leading causes of mortality and morbidity worldwide, and the principal cause of both in Western countries for individuals under the age of 45 [7,8,9,10]. This public health issue therefore places an enormous physical, psychological and socioeconomic burden on healthcare services, individuals and communities around the world [11,12,13]. For instance, in the United States, an annual economic burden of USD 76.5 billion has been estimated for this type of injury, encompassing total lifetime medical costs and productivity losses due to TBI [1,14].

Brain injury results from an external mechanical force to the head, face or neck which leads to a series of complex pathological alterations in neural homeostasis [15]. The most common modes of TBI include falls, motor vehicle accidents, sport-related injuries, military injuries and assaults. Survivors of TBI can experience complications ranging from temporary neurological and psychosocial problems to long-term, severe disability and neurodegenerative disease [13,16,17]. Clinical features may include prolonged coma, seizures, headaches, nausea, aphasia and amnesia, as well as behavioural abnormalities including increased aggression or anxiety. Symptoms can manifest within minutes of injury and may persist for months or years post-TBI [13,18,19]. Currently, there are no known effective treatments for TBI and no FDA-approved therapies to prevent or limit the deficits caused by brain injuries. This is a likely consequence of the heterogeneity of the disease and the many complex biochemical and pathophysiological events which occur at multiple time points following injury, the underlying mechanisms of which are yet to be fully elucidated [7,13,20].

MicroRNAs (miRNAs, miRs) are a class of small, endogenous, non-coding RNA molecules which regulate protein synthesis at the post transcriptional level by binding to the 3′-untranslated region (UTR) of target genes, resulting in translational suppression and mRNA degradation [21,22]. The human genome is believed to encode over 2000 miRNAs, which are estimated to play a role in targeting and regulating the expression of 30–60% of all genes, with single miRNAs possessing the ability to regulate up to several hundred target mRNAs [10,22,23,24]. These molecules therefore control a wide range of biological functions and processes including development, differentiation, apoptosis, immune responses and cell metabolism [20]. In addition, miRNAs are free circulating in the bloodstream as well as in other biofluids (CSF, saliva, urine, etc.), acting as effectors of cell-to-cell communication. They may also be passively released into biofluids as result of cell injury, thereby potentially serving as biomarkers of injury and/or toxicity. Several studies have already demonstrated the utility of microRNAs as potential circulating biomarkers in the context of TBI [1,10,22].

The ability of a single miRNA to regulate up to several hundred target genes makes miRNA particularly suitable for therapeutic purpose. In addition, tissue-specific viral injection, which allows precise spatially targeted therapies and avoids the pitfalls of systematically delivered oligonucleotides, are already in development.

Therefore, in this research, two independent systematic reviews were conducted, investigating both miRNA and proteomic expression in brain tissues of sTBI rat models with the aim to identify novel in situ-targets able to mitigate the devastating effects of secondary brain injury. By combining the two individual reviews, we hoped to establish associations between miRNA and protein expression following sTBI in order to better understand the pathways and underlying mechanisms involved in this disease pathology and to highlight any potential novel targets for the condition.

## 2. Methods

### 2.1. Literature Search

Throughout the conduction of this systematic review, the preferred reporting items for systematic reviews and meta-analysis (PRISMA) statement for systematic reviews was adhered to [25]. Two independent searches of the literature were conducted, the results of which were combined to perform integrated miRNA-protein co-expression analyses. To ensure that the literature was reviewed sufficiently and that all appropriate articles for inclusion were found, 3 independent databases were searched: PubMed, Web of Science and Scopus. The search terms applied to each database for the miRNA and protein searches were ‘traumatic brain injury’, ‘microRNA’ and ‘rat model’, and ‘traumatic brain injury’, ‘proteins’, ‘rat model’ and ‘biomarker’, respectively. Search terms were applied to all fields rather than simply to the title or abstract to ensure all suitable papers were captured in the search. To structure the search, Boolean operators were used as follows: (1) ‘(traumatic brain injury) AND (microRNA) AND (rat model)’ and (2) ‘(traumatic brain injury) AND (proteins) AND (rat model) AND (biomarker)’. Results from each search were copied into separate Microsoft Excel 2016 (Microsoft Corporation, Redmond, WA, USA, available at: https://office.microsoft.com/excel, accessed on 15 June 2021) spreadsheets, including information surrounding the authors, title, publication year, digital object identifier (DOI) and web address for each search result.

### 2.2. Selection Criteria

Two authors (C.O. and V.D.P.) independently scrutinised the titles and abstracts of each paper generated from the literature search to assess their eligibility according to our inclusion and exclusion criteria. The selection criteria for the searches were developed based on the following inclusion (Table 1) and exclusion criteria (Table 2):

### 2.3. Data Collection

The two researchers independently extracted the following study characteristics from the selected, eligible studies for each review: authors, title, year, web address, access to full text, primary paper, English language, animal model used (rat), sample size, injury model used, severity of TBI, methodology of miRNA and protein expression analysis, time point of analysis, results (miRNAs/proteins named). Any additional, useful information regarding study findings or limitations was also recorded.

In order to be able to perform bioinformatic analysis, a separate table for both the miRNA literature search and the protein literature search were created, detailing the names of each miRNA or protein found to be differentially expressed (significantly upregulated, downregulated or both), along with the time point of analysis post-injury and the associated fold changes and *p* values. MiRNAs and proteins that were found to be differentially expressed across 3 or more of the selected papers were recorded. This additional information helped to determine whether it was possible to perform a meta-analysis.

### 2.4. Bioinformatic Analysis

To establish the target genes of the differentially expressed miRNAs found in the literature, DIANA Tools mirPath (DIANA-miRpath v.3, University of Thesselay, Greece, available on line: DIANA TOOLS—mirPath v.3 (grnet.gr), accessed on 15 June 2021) [26] was used and the resulting targets screened to determine any overlap with the proteins also found. An additional search was performed in order to validate miRNA-target interactions and using 2 separate and different tools: miRTargets, (miRDB, MO, USA, available online: http://mirdb.org/, accessed on 15 June 2021) and miRwalk (miRwalk, Heidelberg, DE, available online: http://mirwalk.umm.uni-heidelberg.de/, accessed on 1 Septemeber 2021). The direction of expression fold change for the associated miRNAs and proteins were evaluated, with any inverse relationships being highlighted—this was executed to account for the fact miRNAs inversely regulate protein expression through a negative feedback mechanism [3]. Additionally, these interactions were checked to observe whether they were experimentally validated, using TarBase (Tarbase v8.0, Greece, available online: https://carolina.imis.athena-innovation.gr/diana_tools/web/index.php?r=tarbasev8%2Findex, accessed on 1 September 2021), an online database for functional annotations. For those miRNAs and proteins with an inverse expression relationship, KEGG pathway analysis was performed to identify the biological pathways in which they are implicated. For the miRNAs, analysis was conducted using DIANA Tools mirPath [26], while for the proteins, DAVID tool (DAVID v6.8, MD, USA, available online: (https://david.ncifcrf.gov/content.jsp?file=Acknowledgements.html, accessed on 15 June 2021) [27,28] software was used. Pathways in common for both the miRNAs and proteins were then documented before DIANA Tools mirPath, and the KEGG Pathway Database (KEEG, Kyoto Encyclopedia of Genes and Genomes, Japan, available online: https://www.genome.jp/kegg/pathway.html, accessed on 15 July 2021) was used to reveal the miRNAs and proteins involved in these common pathways.

### 2.5. Risk of Bias

Risk of bias was assessed using the SYRCLE risk of bias (RoB) tool for animal studies [29]. Risk for each study was assessed across 10 domains by two independent reviewers (C.O. and Z.A.), with any disagreements being settled through discussion. The domains used to assess the potential risk of bias across the studies were: random sequence generation, baseline characteristics described, correct timing of randomization, allocation concealment, random housing, blinding, random outcome assessment, incomplete data, sample size calculation and primary outcome specified. Algorithms were then used to determine whether each study had either a low, some, or high risk of bias.

## 3. Results

### 3.1. Study Selection

**Figure 1:**
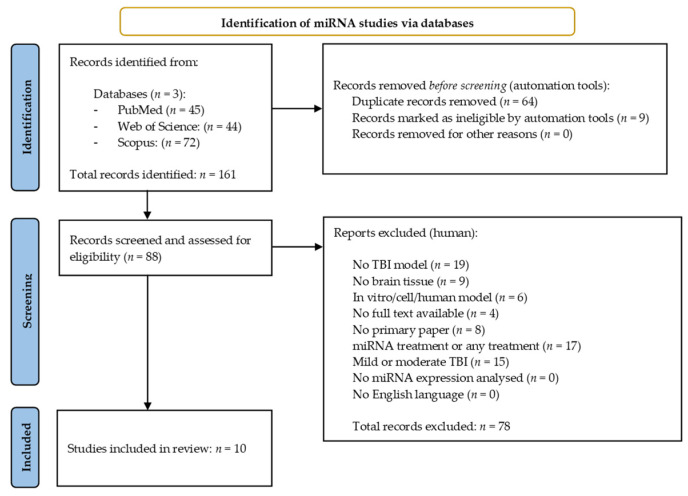
Preferred reporting items for systematic reviews and meta-analysis (PRISMA) flow chart detailing the literature search process for miRNA studies.

The miRNA search yielded 161 results across the three databases. Of these, 64 duplicate studies were identified and excluded using the reference management software, EndNote (EndNote v.20, PA, USA) [30], along with nine papers which were excluded once filters for free full text/open access were applied within the databases themselves. The remaining 88 records were then manually screened and assessed for eligibility against the selection criteria previously described (Section 2.2). Seventy-eight results were excluded during this process, leaving 10 miRNA studies for inclusion in the systematic review [31,32,33,34,35,36,37,38,39,40]. Figure 1 provides an overview of the miRNA literature search and study selection process.

**Figure 2:**
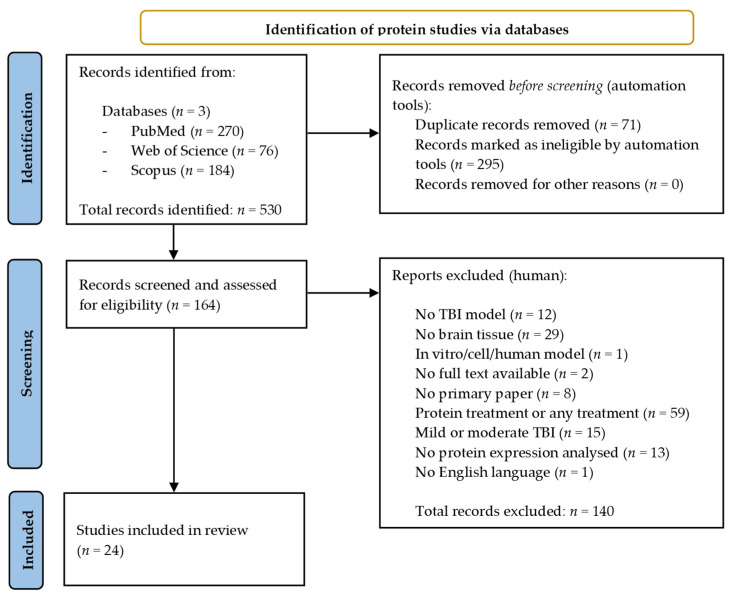
Preferred reporting items for systematic reviews and meta-analysis (PRISMA) flow chart detailing the literature search process for protein studies.

A total of 530 records were identified across the three databases from the protein search. The same search filters were applied, leading to 295 results being excluded while a further 71 duplicates were removed using EndNote 20 [30]. A total of 140 papers were manually excluded following assessment against the selection criteria, leaving a total of 24 papers eligible for inclusion in the review [36,41,42,43,44,45,46,47,48,49,50,51,52,53,54,55,56,57,58,59,60,61,62,63]. Figure 2 provides an overview of the protein literature search and study selection process.

### 3.2. Study Characteristics

All studies included are primary research papers investigating changes in either miRNA or protein expression following severe TBI in rats. The injury model, time point of analysis post-TBI and analysis method differed across the studies. Supplementary Materials Tables S1 and S2 provide an overview of the study characteristics for the included papers [31,32,33,34,35,36,37,38,39,40,41,42,43,44,45,46,47,48,49,50,51,52,53,54,55,56,57,58,59,60,61,62,63]. The data collected here allowed us to establish whether performance of a meta-analysis was possible. In concurrence with a qualified statistician, the conclusion was reached that, due to the heterogeneous nature of data presentation, study techniques and analysis methods across the miRNA and protein studies, completion of a meta-analysis was not possible.

### 3.3. miRNA and Protein Expression Changes Post-TBI

The names of each miRNA and protein found to be differentially expressed in rat brain tissue following severe TBI were extracted from each of the included papers, along with whether expression was significantly up- or downregulated at each time point following injury. A comprehensive list of each differentially expressed miRNA or protein and the direction of change can be found in Tables S1 and S2 in Supplementary Materials, respectively.

### 3.4. Bioinformatic Analysis

To determine the gene targets of each differentially expressed miRNA, DIANA Tools mirPath v.3 software was used. The targets for each were copied and Microsoft Excel 2016 was used to identify any overlaps between those miRNAs targets and the proteins also found to be differentially expressed in the literature. In total, 57 miRNA target genes and proteins were found to overlap (Table S3 in Supplementary Materials). Data previously collected regarding the direction of expression fold change was consulted to uncover which of the overlapping miRNAs and proteins had an inverse expression relationship. Our results showed that expression of 58 miRNAs was inversely correlated to at least one of the proteins, while expression of 46 proteins was inversely correlated to at least one of the miRNAs. This data is illustrated in Table 3. In Table 3, we also show whether these interactions were detected using different prediction tools (miTargets and miRwalk) and whether they were experimentally validated.

### 3.5. KEGG Pathways Analysis to Identify Potential miRNA Processes and Targets for TBI

For those miRNAs and proteins with an inverse expression relationship, KEGG pathway analysis was performed to identify the biological pathways in which they are implicated. For miRNAs, analysis was conducted using DIANA Tools mirPath v.3 [26], and for proteins, DAVID v6.8 [27,28] software was used. A full list of the KEGG pathways identified for the miRNAs and proteins with inverse expression relationships can be found in the Supplementary Materials (Table S4 in Supplementary Materials). Two KEGG pathways were found to be in common between the miRNAs and proteins: endocytosis and TNF signalling. The miRNAs and proteins/genes implicated in these pathways are shown in Table 4.

### 3.6. Risk of Bias (RoB)

**Figure 3:**
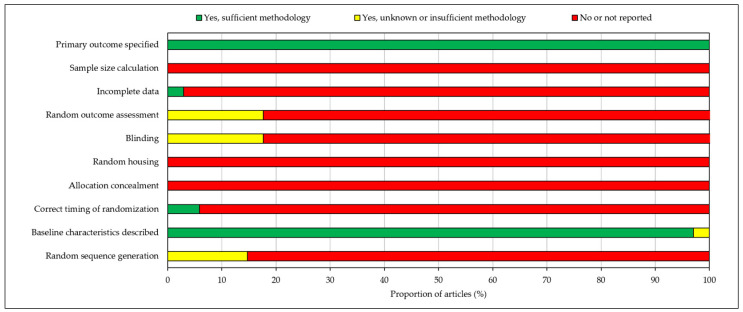
Risk of bias assessment according to the SYRCLE risk of bias (RoB) tool for animal studies. Risk of bias was judged to be high.

RoB analysis found that all included articles specified the primary outcomes and described baseline characteristics. Nearly all studies (97%) had full data availability with no missing data. However, no studies calculated sample size, randomly housed animals or concealed treatment allocations. Incidences of random sequence generation, correct timing of randomisation, blinding and random outcome assessment were also low, with 85–100% of studies reporting no information concerning these domains. As a result, overall risk of bias was judged to be high and hence may invalidate certain findings of the included papers. RoB analysis results are depicted in a stacked bar chart in Figure 3.

## 4. Discussion

This systematic review identified 34 studies (10 miRNA and 14 protein studies) that met our inclusion and exclusion criteria in relation to changes in miRNA and protein expression following TBI in the brain tissue of rats. Due to the heterogeneous nature of parameters including data presentation, study technique and analysis method across those included, meta-analysis was not deemed possible, hence studies were analysed qualitatively. Our results demonstrated that 57 gene targets of the differentially expressed miRNAs identified in the literature overlapped with the proteins also found to be significantly up- or downregulated following sTBI. By studying those overlapping miRNAs and proteins which had inverse expression relationships, we were able to uncover the common KEGG pathways and highlight potential novel targets for therapeutic intervention.

Two KEGG pathways were found to be in common: the endocytosis pathway and TNF signalling pathway. Upon review of the literature, little was found to associate the endocytosis pathway with TBI or any other CNS pathologies. Endocytosis involves the internalisation of cell surface receptors, ligands, nutrients, plasma membrane proteins and lipids, bringing them to the interior of the cell [82]. This process occurs via pinocytosis, phagocytosis or receptor-mediated endocytosis, with cargo progressing through several stages of sorting and routing before being degraded by lysosomes and recycled back to the cell surface or secreted [82,83]. Despite the fact that several endocytic proteins are known to be dysregulated in certain disease states and that functionally active miRs, such as miR-29a and miR-125a, are secreted in the synaptosomes via exocytosis and endocytosis pathways, the involvement of the endocytosis pathway in TBI does not appear to be well documented in the literature [84].

On the contrary, the implication of the TNF signalling pathway in TBI has previously been reported. Several miRs have been already described as regulators of TNF [85,86,87]. TNF is a critical cytokine able to induce a number of important intracellular signalling pathways, including both inflammation and cell death (apoptosis), with dysregulation of its downstream NF-Κb signalling pathway, giving rise to chronic inflammation responsible for several human pathologies [88]. TNFR1 is the principal receptor for TNF and is expressed in almost all cells, while the second receptor, TNFR2, is expressed in a limited range of cells including microglia, oligodendrocytes and specific neuron subtypes [89]. The previously documented involvement of this pathway in inflammation and apoptosis processes, as well as the CNS-specific expression of its receptors, allows us to be confident when speculating the involvement of this pathway in the pathogenesis of TBI and other CNS diseases as our results would suggest.

Evidence exists which indicates that TNF signalling plays an important role in exacerbating both Amyloid-β (Aβ) and tau pathologies in vivo [90]. APP (amyloid precursor protein) and MAPT (microtubule associated protein tau) were both found to exhibit an inverse expression relationship with multiple miRNAs in this systematic review. In our results, APP expression was shown to be inversely correlated with that of miR-185-5p and miR-144-3p, and MAPT with miR-671-5p and miR-298-5p. Wu et al. have reported that, through a series of signalling cascades, TBI results in the cleavage of both APP and tau (at APP N585a and Tau N368 sites, respectively), mediating Alzheimer’s disease pathogenesis through the promotion of Aβ production and tau hyperphosphorylation, processes which ultimately induce neuroinflammation and neurotoxicity [91]. Furthermore, the presence of extracellular Aβ plaques has been recorded both acutely and chronically following severe TBI, and similar aggregates are also found in elderly people of normal cognitive status [92,93,94]. The involvement of APP and MAPT in certain neurodegenerative diseases, ageing, and most importantly, in TBI is well documented and supportive of our findings. Therefore, targeting miRNAs which negatively regulate TNF signalling or APP expression directly may be used as strategy to prevent accumulation of this protein and subsequent neurological disease.

Our current knowledge of miRNAs and their roles in several physiological and pathological processes have led to them serving as important emerging candidates for novel therapeutic targets in CNS injury [95]. These molecules possess numerous characteristics which make them extremely attractive tools for therapeutic intervention; in addition to the previously described knowledge that manipulation of one miRNA can affect many targets, certain other desirable features have contributed to the increasing interest surrounding these molecules in terms of drug development [95,96]. For example, not only does their short length (~22 nucleotides) allow miRNA-based drugs to be easily designed; they are also frequently conserved between species, and several drug delivery systems have been approved for human use which allow in vivo delivery of these emerging therapeutics [95,97,98,99]. Examples of miRNA-based therapeutics are miRNA mimics (agomir) and miRNA inhibitors (antagomiRs), which decrease and increase target gene expression, respectively [95]. As the name suggests, miRNA mimics and can act as compensatory agents in the event of a loss of miRNA functional activity due to downregulation of expression in correlation with disease progression. Conversely, in a situation where specific miRNAs are upregulated in response to injury and appear to be contributing towards disease pathogenesis, miRNA inhibitors can be used with the aim of suppressing this overexpression [95].

It is possible to reliably predict potential gene targets based on the partially complementary sequences between mature miRNAs and mRNA candidate targets. As a result, several pharmaceutical companies have been investigating the application of miRNAs as therapeutics in recent years, with various drugs advancing to human trial stages, such as Miravirsen, RG-101, RG-125/AZD4076, MRX34, and TagomiRs [95,99,100,101,102]. However, despite certain miRNA drug candidates displaying promising effects and an ability to improve neurological deficits post-injury in preclinical studies, phase I and phase II trials, of the more than 30 clinical trials which have taken place over the past 3 decades, all candidates have failed to demonstrate therapeutic efficacy in humans with TBI [7,13,103]. This widespread failure may be attributed to the heterogeneity of this form of injury, as well as the variability in treatment protocols between trial sites [7]. Regardless of the reason, this lack of success has highlighted the urgent need for novel target discovery for this increasingly devastating disease.

### Limitations

One of the main limitations of this study is the variation in time point of expression analysis from the initial time of injury (ranging from 2 h to 12 months post-TBI). Given that biomarker signatures are constantly changing and evolving following a brain injury, this variation in analysis time point makes comparisons between studies extremely difficult. Another significant limitation of this study is that included studies used different models of TBI, including the fluid-percussion, cortical impact, penetrating ballistic-like brain injury models, which may affect different microRNAs due to the different mechanisms of injury. Certain studies also used laser capture microdissection, whilst the majority of the studies used specific regions of the brain to harvest mRNA and protein, potentially introducing greater variety. Other limitations include the heterogeneity between studies in terms of data presentation, study technique and analysis method, which rendered the performance of a meta-analysis impossible. Furthermore, studies failed to report sample sizes; this along with a number of other shortcomings meant that overall risk of bias was judged to be high. High risk of bias should be avoided going forward through adherence to guidelines outlining standardised techniques for animal studies such as the ARRIVE (Animal Research: Reporting of In Vivo Experiments) guidelines [104]. In the future, investigators should focus efforts towards identifying clear miRNA and/or protein biomarker signatures across a set of reasonable time points, reducing variation in study techniques and analysis methods, all while ensuring adequate sample sizes are used and appropriate guidelines are adhered to.

## 5. Conclusions

In part, our results support the existing literature surrounding the involvement of the TNF signalling pathway, APP, MAPT and their associated miRNAs in the pathogenesis of TBI and other neuropathological conditions, and hence their potential as therapeutic targets. However, the involvement of the endocytosis pathway in this field of research has yet to be widely discussed. Although we can speculate involvement of both biological pathways in TBI, the limitations discussed above as well as the high risk of bias and lack of meta-analysis are considerably limiting factors in this review, hence caution is advised when interpreting this data and further high-quality studies are required. In recent years, miRNAs have shown immense potential as diagnostic biomarkers and therapeutic targets in a number of pathologies, including TBI. Further studies are needed in both animal and human models of TBI to better understand the mechanisms underlying the pathogenesis of this increasingly devastating disease, the miRNA-protein interactions which occur at different time points following brain injury, and to ultimately uncover the most effective targets for treatment.

## Figures and Tables

**Table 1:** Inclusion criteria.

Protein Studies to Be Included If:	miRNAs—Studies to Be Included If:
Severe TBI model	Severe TBI model
Rat brain tissue	Rat brain tissue
Full text available	Full text available
Primary research paper	Primary research paper
Protein expression analysed	miRNA expression analysed
English language	English language

**Table 2:** Exclusion criteria.

Proteins—Studies Excluded If:	miRNAs—Studies Excluded If:
No TBI model	No TBI model
No brain tissue	No brain tissue
In vitro/cell/human model utilised	In vitro/cell/human model utilised
No full text available	No full text available
No primary paper	No primary paper
Protein expression after any treatment	miRNA expression after any treatment
Mild or moderate TBI	Mild or moderate TBI
Protein expression not analysed	miRNA expression not analysed
No English language	No English language

**Table 3:** miRNA-protein interactions predicted by DIANA tools and confirmed by miRtaregts and miRwalk tools. The table also reports the inverse expression relationships between miRNAs and proteins and whether the interaction was experimentally validated.

Protein Target	miRNA Interaction (DIANA-Tools)	miRNA Interaction (miRTargets)	miRNA Interaction (miRwalk)	miRNA Expression	Protein Expression	Inverse Expression	Experimentally Validated
FOXJ1	miR-200a-3p	x	x	↑	↑	N	
	miR-325-3p		x	↓	↑	Y	
CXCL1	miR-150-5p		x	↓	↑	Y	
CCL2	miR-369-3p			↓	↑	Y	
CCL20	miR-221-3p			↓	↑	Y	
	miR-376c-3p			↓	↑	Y	
ACO1	miR-200a-3p		x	↑	↑	N	
	miR-223-3p			↑	↑	N	
C3	miR-127-5p	x	x	↓	↑	Y	
GMPS	miR-23a-3p			↑	↑	N	[64]
	miR-23b-3p		x	↑	↑	N	[65]
	miR-200a-3p		x	↑	↑	N	
	miR-200b-3p		x	↑	↑	N	
	miR-200c-3p		x	↑	↑	N	
	miR-224-5p		x	↑	↑	N	[66]
	miR-543-3p			↓	↑	Y	
DPYSL2	miR-29c-3p	x		↓	↑ and ↓	Y	
	miR-29a-3p	x	x	↓	↑ and ↓	Y	
	miR-30a-5p	x		↑	↑ and ↓	Y	
	miR-30b-5p	x	x	↑	↑ and ↓	Y	
	miR-30c-5p	x	x	↑ and ↓	↑ and ↓	Y	
	miR-30e-5p	x		↑	↑ and ↓	Y	
	miR-130a-3p	x	x	↓	↑ and ↓	Y	
	miR-130b-3p	x		↑	↑ and ↓	Y	
	miR-140-5p	x	x	↓	↑ and ↓	Y	
	miR-181b-5p		x	↓	↑ and ↓	Y	
	miR-224-5p	x	x	↑	↑ and ↓	Y	
	miR-329-3p	x	x	↓	↑ and ↓	Y	
	miR-721			↑	↑ and ↓	Y	
DPYSL3	miR-132-3p	x		↑	↑	N	[64]
	miR-212-3p	x	x	↓	↑	Y	
DPYSL5	miR-19a-3p	x	x	↓	↑	Y	
	miR-19b-3p	x	x	↑ and ↓	↑	Y	[67]
	miR-20b-5p	x	x	↑	↑	N	
	miR-29a-3p	x		↓	↑	Y	
	miR-29c-3p	x		↓	↑	Y	
	miR-139-5p	x	x	↓	↑	Y	
	miR-153-3p	x	x	↑	↑	N	
	miR-224-5p		x	↑	↑	N	
	miR-342-5p	x	x	↓	↑	Y	[68]
	miR-467a-5p			↑	↑	N	
	miR-667-3p			↓	↑	Y	
WDR1	miR-19a-3p	x		↓	↑	Y	
	miR-19b-3p	x		↑ and ↓	↑	Y	
	miR-125a-5p	x		↓	↑	Y	
	miR-125b-5p	x		↓	↑	Y	
BASP1	miR-200b-3p	x		↑	↑	N	
	miR-200c-3p	x	x	↑	↑	N	
	miR-325-3p			↓	↑	Y	
	miR-381-3p		x	↑	↑	N	
CFH	miR-136-5p		x	↓	↑	Y	
	miR-181b-5p		x	↓	↑	Y	
ACO2	miR-744-5p		x	↑	↓	Y	
PLCB1	miR-20b-5p	x	x	↑	↓	Y	
	miR-130a-3p	x		↓	↓	N	
	miR-130b-3p	x		↑	↓	Y	
	miR-139-5p	x	x	↓	↓	N	[64]
	miR-144-3p	x		↓	↓	N	
	miR-153-3p	x	x	↑	↓	Y	
	miR-181a-5p			↓	↓	N	
	miR-181b-5p		x	↓	↓	N	
	miR-325-3p			↓	↓	N	
	miR-499-5p	x		↑	↓	Y	
	miR-721			↑	↓	Y	
	miR-744-5p		x	↑	↓	Y	
UBA1	miR-325-3p			↓	↓	N	
STXBP1	miR-674-5p			↓	↓	N	
STMN1	miR-9-5p	x	x	↑	↓	Y	[69]
	miR-221-3p	x	x	↓	↓	N	
	miR-222-3p	x		↓	↓	N	[69]
SPTBN1	miR-135a-5p			↓	↓	N	
	miR-135b-5p		x	↓	↓	N	
	miR-298-5p			↑	↓	Y	
	miR-320-3p			↓	↓	N	
	miR-671-5p	x	x	↑	↓	Y	
ARF3	miR-329-3p		x	↓	↑	Y	
OXCT1	miR-185-5p		x	↓	↑	Y	
MDH1	miR-25-3p			↓	↑	Y	[66]
	miR-142a-5p			↑	↑	N	
	miR-674-5p			↓	↑	Y	
	miR-691			↑	↑	N	
APP	miR-20b-5p	x	x	↑	↑	N	
	miR-144-3p	x		↓	↑	Y	
	miR-153-3p	x		↑	↑	N	
	miR-185-5p	x	x	↓	↑	Y	
GDI1	miR-150-5p	x	x	↓	↑	Y	
	miR-325-3p			↓	↑	Y	
	miR-329-3p		x	↓	↑	Y	
SPTAN1	miR-29a-3p	x		↓	↑	Y	[70,71,72]
	miR-29c-3p	x		↓	↑	Y	[66,70,71,72]
	miR-325-3p			↓	↑	Y	
ANXA11	miR-124-3p	x	x	↑ and ↓	↓	Y	[73]
ACSS2	miR-125a-5p		x	↓	↓	N	
	miR-125b-5p			↓	↓	N	
PGK2	miR-499-5p			↑	↓	Y	
PGK1	miR-34c-5p		x	↑	↑ and ↓	Y	
	miR-449b		x	↑	↑ and ↓	Y	
GLUD1	miR-379-5p			↓	↓	N	
ALDOA	miR-34c-5p	x	x	↑	↓	Y	
	miR-449b	x		↑	↓	Y	
DDAH1	miR-30a-5p	x		↑	↓	Y	[67,74,75]
	miR-30b-5p	x		↑	↓	Y	[66,67,74,75]
	miR-30c-5p	x		↑ and ↓	↓	Y	[66,67,74,75]
	miR-30e-5p	x		↑	↓	Y	[66,67,74,75]
MAP2	miR-34b-3p	x	x	↑ and ↓	↓	Y	
	miR-129-5p	x	x	↓	↓	N	
	miR-185-5p		x	↓	↓	N	
	miR-200b-3p	x	x	↑	↓	Y	
	miR-200c-3p	x		↑	↓	Y	
	miR-325-3p	x		↓	↓	N	
	miR-335-5p			↓	↓	N	[76]
	miR-361-5p		x	↓	↓	N	[64]
	miR-369-3p	x		↓	↓	N	[64]
	miR-667-3p			↓	↓	N	
NRGN	miR-23a-3p			↑	↑	N	[75]
	miR-23b-3p			↑	↑	N	[75]
	miR-181a-5p			↓	↑	Y	
	miR-181b-5p		x	↓	↑	Y	
	miR-330-5p		x	↓	↑	Y	
PRDX2	miR-325-3p			↓	↑	Y	
SYN2	miR-25-3p	x	x	↓	↑	Y	
	miR-325-3p			↓	↑	Y	
	miR-363-3p	x		↑	↑	N	
	miR-495-3p		x	↓	↑	Y	
HIBADH	miR-132-3p		x	↑	↓	Y	
	miR-212-3p		x	↓	↓	N	
ACTA1	miR-155-5p	x	x	↑	↓	Y	
ARF1	miR-153-3p	x		↑	↓	Y	
	miR-320-3p	x		↓	↓	N	
	miR-342-5p		x	↓	↓	N	
	miR-381-3p	x	x	↑	↓	Y	
	miR-674-5p			↓	↓	N	
AMPH	miR-153-3p	x	x	↑	↓	Y	
	miR-705			↑	↓	Y	
COPS2	miR-103-3p			↓	↓	N	
	miR-107-3p			↓	↓	N	
	miR-181a-5p	x	x	↓	↓	N	[64,66,67,70,75,77,78]
	miR-181b-5p	x	x	↓	↓	N	[64,66,67,70,75,77,78,79]
	miR-200b-3p	x		↑	↓	Y	[71]
	miR-200c-3p	x	x	↑	↓	Y	[71]
	miR-320-3p	x		↓	↓	N	
	miR-674-5p			↓	↓	N	
GAPDH	miR-325-3p			↓	↓	N	
HSPH1	miR-200b-3p			↑	↓	Y	
	miR-200c-3p		x	↑	↓	Y	
	miR-369-3p	x		↓	↓	N	[64]
	miR-667-3p			↓	↓	N	
HSPA4	miR-495-3p		x	↓	↓	N	
MAPT	miR-298-5p			↑	↓	Y	
	miR-433-3p		x	↓	↓	N	
	miR-671-5p		x	↑	↓	Y	
NLN	miR-144-3p			↓	↓	N	
	miR-325-3p			↓	↓	N	
NDRG2	miR-325-3p			↓	↓	N	
PCNP	miR-181a-5p	x	x	↓	↓	N	
	miR-181b-5p	x		↓	↓	N	
	miR-325-3p	x		↓	↓	N	
	miR-495-3p			↓	↓	N	
PDCD6IP	miR-9-5p	x		↑	↓	Y	[64]
	miR-142a-5p	x		↑	↓	Y	
	miR-181b-5p	x	x	↓	↓	N	
PDHA1	miR-34b-3p		x	↑ and ↓	↓	Y	
	miR-381-3p			↑	↓	Y	
RAB3C	miR-25-3p	x	x	↓	↓	N	
	miR-34c-5p		x	↑	↓	Y	
	miR-325-3p			↓	↓	N	
	miR-329-3p			↓	↓	N	
	miR-335-5p			↓	↓	N	[76]
	miR-363-3p	x	x	↑	↓	Y	
	miR-369-3p			↓	↓	N	
	miR-495-3p	x		↓	↓	N	[64]
PPP3CC	miR-382-5p	x	x	↓	↓	N	
SNAP25	miR-130a-3p	x	x	↓	↓	N	[71]
	miR-130b-3p	x	x	↑	↓	Y	[71]
	miR-153-3p		x	↑	↓	Y	[80]
	miR-185-5p	x	x	↓	↓	N	
	miR-200b-3p	x		↑	↓	Y	
	miR-200c-3p	x		↑	↓	Y	
	miR-221-3p			↓	↓	N	
	miR-222-3p			↓	↓	N	
	miR-721			↑	↓	Y	
TAGLN3	miR-153-3p	x		↑	↓	Y	
BACE1	miR-9-5p	x	x	↑	↑	N	[81]
	miR-19a-3p	x		↓	↑	Y	
	miR-19b-3p	x	x	↑ and ↓	↑	Y	
	miR-103-3p			↓	↑	Y	
	miR-107-3p			↓	↑	Y	
	miR-124-3p	x	x	↑ and ↓	↑	Y	
	miR-135a-5p	x	x	↓	↑	Y	
	miR-135b-5p	x	x	↓	↑	Y	
OAT	miR-181b-5p		x	↓	↑	Y	
	miR-369-3p			↓	↑	Y	
SLC23A2	miR-127-5p		x	↓	↑	Y	
	miR-139-5p		x	↓	↑	Y	
	miR-142a-5p			↑	↑	N	
	miR-144-3p	x		↓	↑	Y	
	miR-200b-3p	x		↑	↑	N	
	miR-200c-3p	x	x	↑	↑	N	
	miR-382-5p			↓	↑	Y	
	miR-665-3p	x		↑	↑	N	

**Table 4:** miRNAs and proteins implicated in the Endocytosis and TNF signalling KEGG pathways.

TNF Signalling Pathway		Endocytosis Pathway	
miRNAs Involved (*n* = 53):	Proteins/Genes Involved (*n* = 55):	miRNAs Involved (*n* = 55):	Proteins/Genes Involved (*n* = 129):
miR-103-3p	Akt1	miR-103-3p	2610002M06Rik
miR-107-3p	Atf2	miR-107-3p	Acap2
miR-124-3p	Bag4	miR-124-3p	Acap3
miR-125a-5p	Bcl3	miR-125a-5p	Adrb1
miR-125b-5p	Ccl2	miR-125b-5p	Adrb2
miR-130a-3p	Ccl20	miR-127-5p	Adrb3
miR-130b-3p	Cebpb	miR-130a-3p	Adrbk1
miR-135a-5p	Cflar	miR-130b-3p	Agap1
miR-135b-5p	Chuk	miR-132-3p	Ap2b1
miR-136-5p	Creb1	miR-135a-5p	Ap2m1
miR-139-5p	Creb3l1	miR-135b-5p	Arap2
miR-140-5p	Creb3l2	miR-136-5p	Arap3
miR-142a-5p	Creb5	miR-139-5p	Arf3
miR-144-3p	Csf1	miR-140-5p	Arf5
miR-150-5p	Cx3cl1	miR-142a-5p	Arf6
miR-153-3p	Cxcl1	miR-144-3p	Arfgef1
miR-155-5p	Cxcl10	miR-150-5p	Arfgef2
miR-181a-5p	Dnm1l	miR-153-3p	Arrb1
miR-181b-5p	Edn1	miR-155-5p	Asap1
miR-185-5p	Fos	miR-181a-5p	Asap2
miR-19a-3p	Il18r1	miR-181b-5p	Cav1
miR-19b-3p	Jun	miR-185-5p	Cav2
miR-200b-3p	Junb	miR-19a-3p	Cbl
miR-200c-3p	Lif	miR-19b-3p	Cblb
miR-20b-5p	Lta	miR-200b-3p	Cdc42
miR-221-3p	Magi2	miR-200c-3p	Chmp1a
miR-224-5p	Map2k1	miR-20b-5p	Chmp2b
miR-25-3p	Map2k4	miR-212-3p	Chmp3
miR-298-5p	Map2k7	miR-221-3p	Chmp4c
miR-29a-3p	Map3k14	miR-224-5p	Chmp5
miR-29c-3p	Map3k5	miR-25-3p	Chmp6
miR-30a-5p	Map3k7	miR-298-5p	Chmp7
miR-30b-5p	Map3k8	miR-29a-3p	Clta
miR-30c-5p	Mapk10	miR-29c-3p	Cltb
miR-30e-5p	Mapk12	miR-30a-5p	Cltc
miR-325-3p	Mapk14	miR-30b-5p	Cxcr2
miR-329-3p	Mapk8	miR-30c-5p	Cxcr4
miR-330-5p	Mapk9	miR-30e-5p	Cyth1
miR-342-5p	Nfkb1	miR-325-3p	Cyth3
miR-363-3p	Nfkbia	miR-329-3p	Dab2
miR-369-3p	Pik3cb	miR-330-5p	Dnm1
miR-376c-3p	Pik3cd	miR-363-3p	Dnm3
miR-381-3p	Pik3r1	miR-369-3p	Eea1
miR-382-5p	Pik3r2	miR-376c-3p	Egfr
miR-495-3p	Pik3r3	miR-381-3p	Ehd2
miR-499-5p	Rela	miR-382-5p	Ehd3
miR-543-3p	Rps6ka4	miR-495-3p	Ehd4
miR-667-3p	Rps6ka5	miR-499-5p	Epn2
miR-671-5p	Socs3	miR-543-3p	Epn3
miR-674-5p	Tab2	miR-667-3p	Eps15
miR-705	Tab3	miR-671-5p	Erbb4
miR-721	Tnf	miR-674-5p	F2r
miR-9-5p	Tnfrsf1a	miR-705	Fgfr2
	Tnfrsf1b	miR-721	Flt1
	Traf3	miR-9-5p	Folr2
			Gbf1
			Git2
			Grk1
			Grk4
			Grk5
			H2-K1
			H2-M3
			H2-Q1
			H2-T23
			Hspa1b
			Hspa2
			Igf1r
			Iqsec1
			Iqsec2
			Kdr
			Kit
			Ldlrap1
			Mdm2
			Met
			Nedd4
			Nedd4l
			Pard3
			Pard6b
			Pdcd6ip
			Pip5k1b
			Pip5k1c
			Pld1
			Pld2
			Pml
			Prkcz
			Psd
			Psd2
			Psd3
			Rab11a
			Rab11b
			Rab11fip1
			Rab11fip2
			Rab11fip4
			Rab11fip5
			Rab22a
			Rab5a
			Rab5b
			Rab5c
			Ret
			Rhoa
			Sh3glb1
			Sh3kbp1
			Smad6
			Smad7
			Smap1
			Smap2
			Smurf1
			Smurf2
			Src
			Stam
			Stam2
			Tfrc
			Tgfb2
			Tgfbr1
			Tgfbr2
			Tonsl
			Traf6
			Usp8
			Vps25
			Vps36
			Vps37a
			Vps37b
			Vps37c
			Vps37d
			Vps4b
			Wwp1
			Zfyve16
			Zfyve20
			Zfyve9

## Data Availability

All data generated as part of this study are included in the article.

## Supplementary Materials

The following are available online at https://www.mdpi.com/article/10.3390/cells10092425/s1, Table S1: Study characteristics and differentially regulated miRNAs post-sTBI, Table S2: Study characteristics and differentially regulated proteins post-sTBI, Table S3: Common miRNA targets and differentially expressed proteins, Table S4: KEGG pathways identified for miRNAs and proteins with inverse expression relationships and their significance (*p*-value). Pathways highlighted in blue represent common pathways.
