# Supplementary material for: Co-Expression Network Analysis of MicroRNAs and Proteins in Severe Traumatic Brain Injury: A Systematic Review
Source: Cells. 2021 Sep 14;10(9):2425. doi: 10.3390/cells10092425 (PMC8465595; doi:10.3390/cells10092425)
Supplement: Supplementary file 1 [file cells-10-02425-s001.zip › cells-1363589-supplementary.pdf]

SUPPLEMENTARY MATERIALS

Co-expression network analysis of microRNAs and proteins in severe traumatic brain injury: a systematic review

Claire Osgood [1], Zubair Ahmed [1,2,3*] and Valentina Di Pietro [1,2,3*]

1 Neuroscience and Ophthalmology, Institute of Inflammation and Ageing, University of Birmingham, Edgbaston, Birmingham, B15 2TT, UK;

CXO738@student.bham.ac.uk

2 Centre for Trauma Sciences Research, Edgbaston, Birmingham, B15 2TT, UK;

3 Surgical Reconstruction and Microbiology Research Centre, National Institute for Health Research, Queen Elizabeth Hospital, Birmingham, B15 2TH, UK;

* Correspondence: z.ahmed.1@bham.ac.uk (Z.A.); v.dipietro@bham.ac.uk (V.D.P).

Supplementary Materials

Table S1. Study characteristics and differentially regulated miRNAs post-sTBI.

Table S2. Study characteristics and differentially proteins post-sTBI.

Table S3. Common miRNAtargets and differentially expressed proteins.

Table S4. KEGG pathways identified for miRNAs and proteins with inverse expression relationships and their significance (p-value). Pathways highlighted in blue represent common pathways.

Table S1. Study characteristics and miRNAs differentially regulated in post-sTBI rat brains.

Reference; year of publication; injury model (CCI = controlled cortical impact, FPI = fluid percussion injury, PBBI = penetrating ballistic-like brain injury); brain site; Time point groups (Time <3 d , 3d<Time <2w , Time >1m); methodology (NGS = next generation sequencing, qRT-PCR = real-time quantitative reverse transcription polymerase chain reaction, qPCR = quantitative polymerase chain reaction).

Study	Year	Injury Model	Brain site	Time <3 d	3d<Time <2w	Time >1m	Methodology
Hu et al.[31]	2012	CCI	hippocampus	**24 h ↑:**<br>miR-200a, miR-200b, miR-439, miR23a, miR-873, miR-153, miR-499,miR-381<br>**24h ↓:**<br>miR-31, miR-541, miR-598-5p, miR-19a, miR-19b, miR-222, miR-296, miR-708, miR-144, miR-341, miR-136, miR-148-5p, miR-342-5p	**7 d ↑:**<br>miR-23a, miR-363 and miR-130b<br>**7d ↓:**<br>miR-190, miR-135b, miR-342-5p, miR-135a, miR-148b-5p, miR136 and miR-144		NGS
Jadhav et al.[32]	2014	Rodent FPI	forebrain	**24h ↓:**<br>miR-200b			qRT-PCR
Korotkov et al.[33]	2020	Lateral FPI	cortex		**2w ↑:**<br>miR-142, miR-155		qRT-PCR
Puhakka et al.[34]	2017	Lateral FPI	dentate gyrus			**3m ↓:**<br>miR-369-3p, miR-384-3p, miR136-5p, miR-127-3p, miR-136-3p, miR-335, miR-376a-3p, miR-551b3p, miR-341, miR-139-5p, miR127- 5p and miR-9a-3p	qRT-PCR
Redell et al.[35]	2009	CCI	hippocampus	**3h ↑:**<br>miR-214, miR-298, miR-30a-3p, miR30a-5p, miR-30c, miR-467b, miR-665, miR-675-5p, miR-691, miR-721, miR327<br>**24h ↑:**<br>miR-126-3p, miR-155, miR-19b, miR-21, miR-223, miR-292-5p, miR-9, miR-92<br>**3h and 24h ↑:**<br>miR-23a, miR-23b, miR-290, miR-30b, miR-30e, miR-451, miR-467a, miR-671, miR-680, miR-685, miR-689, miR-705, miR-711, miR-714, miR-744, miR-762<br>**3h ↓:**<br>miR-125a, miR-125b, miR-127, miR-146b, miR-148a, miR-148b, miR-181a, miR-22, miR-221, miR25, miR-320, miR-325, miR-346, miR-376a, miR-485-5p, miR-652, miR-674, miR-690, miR-99b, miR-221<br>**24h ↓:**<br>miR-129-3p, miR-328, miR-361 and miR-487b<br>**3h and 24h ↓:**			qRT-PCR

				miR-103, miR-107,<br>miR-130a, miR-140, miR-150,<br>miR151, miR-181b, miR-185,<br>miR-191, miR-222, miR-323,<br>miR-329, miR-330, miR-379,<br>miR-382, miR-383, miR-422b,<br>miR-433-3p, miR-495,<br>miR-541, miR-543, miR-667,<br>miR140 and miR-151			
Thangavelu et al.[36]	2020	PBBI	coronal brain	**1d ↑:**<br>miR-21, miR-34b, miR-223, miR-1274a, miR-155<br>**3d ↑:**<br>miR-451, miR-685<br>**1d ↓:**<br>miR-19a, miR-327, miR-135a<br>**3d ↓:**<br>miR-328, miR-34b, miR-335,<br>miR-329, miR-667	**7 d ↑:**<br>miR-298, miR-466c, miR-130b,<br>miR-146a, miR-503, miR-224,<br>miR-18a, miR-450, miR-363,<br>miR-142, miR-28, miR-31,<br>miR-10a, miR-21, miR-20b,<br>miR-147b, miR-449, miR-18a,<br>miR-200c, miR-449b, miR-34c,<br>miR-199b, miR-199a, miR-374,<br>miR-155, miR-214, miR-196c,<br>miR-223<br>**7d ↓:**<br>miR-411, miR-667, miR-136,<br>miR-335, miR-434, miR-328,<br>miR181a, miR-376c, miR-409,<br>miR-802, miR-29c, miR-139		qPCR
Vuokila et al.[37]	2020	Lateral FPI	cortex		**7d ↓:**<br>miR-124-3p	**3m ↓:**<br>miR-124-3p	qRT-PCR
Vuokila et al.[38]	2018	Lateral FPI	dentate gyrus			**3m ↓:**<br>miR-124-3p	qRT-PCR
Wang et al.[39]	2021	CCI	hippocampus	**1d ↑:**<br>miR-142-3p, 142-5p, miR-146a,<br>miR-155, miR-223, miR-19b<br>**3d ↑:**<br>miR-142-3p, miR-142-5p, miR-146a, miR-155, miR-223, miR-19b<br>**1d ↓:**<br>miR-124, miR-150<br>**3d ↓:**<br>miR-124, miR-150	**7d ↑:**<br>miR-124,155<br>**7d ↓:**<br>miR-142-3p, miR-142-5p,<br>miR146a, miR-150, miR-22 and<br>miR19b		qRT-PCR
Xiao et al.[40]	2020	Lateral FPI	hippocampus	**3h, 6h, 12h, 24h and 48h ↑:**<br>miR-9a-3p, miR-21-5p,<br>miR-132-3p, miR-219-5p<br>**3h, 6h, 12h, 24h and 48h ↓:**<br>miR-9a-5p, miR-125b-5p,<br>miR347, miR-30c-5p, miR-212-3p, miR-29a-3p			qRT-PCR

Table S2. Study characteristics and proteins differentially regulated in post-sTBI rat brains.

Reference; year of publication; injury model (CCI = controlled cortical impact, FPI = fluid percussion injury, PBBI = penetrating ballistic-like brain injury); brain site; Time point groups (Time <3 d , 3d<Time <2w , Time >1m); methodology (WB = Western Blot, qRT-PCR = real-time quantitative reverse transcription polymerase chain reaction, qPCR = quantitative polymerase chain reaction, ICH= Immunohistochemistry).

Study	Year	Injury Model	Brain site	<3 d	3d<Time <2w	>1m	Methodology
Anderson et al.[41]	2008	CCI	brainstem, cortex, hippocampus	**6h, 24h, 48h ↑:** pNF-H			ELISA
Anwer et al.[42]	2020	Latera l FPI	hypothalamus	**2h, 48h ↓:** SRPX2	**14d ↓:** SRPX2		WB
Bonneh-Barkay et al.[43]	2010	CCI	parietal cortex	**1d, 2d, 3d ↑:** KL-40	**4d, 6d, 8d, 12d ↑:** KL-40		qPCR
Cui et al.[44]	2011	Micro knife injury	cortex	**12h, 1d, 3d ↑:** Foxj1	**5d, 7d ↑:** Foxj1	**14d, 28d ↑:** Foxj1	WB
Dalgard et al.[45]	2012	CCI	cortex	**4h, 12h, 24h, 3d ↑:** CXCL1, IFN-γ, TNF-α, IL-1β, IL-4, IL13, CCL2, CCL20	**7d ↑:** CXCL1, IFN-γ, TNF-α, IL-1β, IL-4, IL13, CCL2, CCL20		ELISA
Das Gupta et al.[46]	2019	Latera l FPI	cortex, thalamus			**7d-1m, 14d-12m ↑:** CLU	qRT-PCR
Dawish et al.[47]	2012	CCI	hippocampus			**25d ↓:** SYP	IHC
DeDominicis et al.[48]	2018	PBBI	hippocampus, striatum, cortex, midbrain	**24h ↑:** GFAP, SBDP-145/150, **24h ↓:** SPTN1	**7d ↑:** SBDP-145/150	**1m, 3m ↑:** GFAP **3m ↓:** SPTN1	WB
Kilbourne et al.[49]	2009	Maryl and model	cortex, corpus callosum, caudate, putamen, thalamus, cerebellum, and brainstem	**1d ↑:** β-APP	**7d ↑:** β-APP		IHC

Kobeissy et al.[50]	2016	CCI	cortex	**1d ↑:**<br>PIN1, SRPRB, EEF2, ACO1, C3, C3 (fragment), GMPS, HSC70, DPYSL2, DPYSL3, DPYSL5, PKM1/M2, WDR1, A1i3, Mug1, ALB, CKBB, PACSIN, ENO2, BASP1, KKBP1A, HbB, VBS, CFH, ENO1, PDI<br>**1d ↓:**<br>ACO2, MnSOD, TPI1, GST (fragment), GSTM1, DJ-1, MMIF, PLCB1, UBA1, NF, CALR, CaM, STXBP1, ENO1, FH, STMN1, CFL1	**7d ↑:**<br>PIN1, SRPRB, EEF2, ACO1, C3, C3 (fragment), GMPS, HSC70, DPYSL2, DPYSL3, DPYSL5, PKM1/M2, WDR1, A1i3, Mug1, ALB, CKBB, PACSIN, ENO2, BASP1, AnxA5, LDH-B, CRYM.<br>**7d ↓:**<br>ACO2, MnSOD, TPI1, GST (fragment), GSTM1, DJ-1, MMIF, PLCB1, UBA1, NF, CALR, CaM and STXBP1.	WB
Kobeissy et al.[51]	2015	CCI	cortex	**2h, 6h, 24h, 3d, ↑:**<br>βsBDPs<br>**2h, 6h, 24h, 3d ↓:**<br>SPTBN1	**5d, 7d, 14d ↑:**<br>βsBDPs<br>**5d, 7d, 14d ↓:**<br>SPTBN1	WB
Kobeissy et al.[52]	2006	CCI	cortex	**48h ↑:**<br>CA1, ARF3, TF, GFER, HbA, HbB, FETUB, OXCT1, MDH1, NAD, LDH-B, MDH2, CES1, SERPINA1, HP, UCLH1, SERPINA1, SERPINA2, T-KNG, ALB, APP, GC, GDI1, CRMP-2, CP, SPTAN1, CRP, PSMA7, SYT<br>**48h ↓:**<br>ANXA11, ALDH7A1, CFL1, PFN1, PKM2, ENO1, GAPDH, HK1, ACSS2, PGK2, PGK1, Hsc70-ps1, GLUD1, ALDOA, ALDOC, DDAH1, MAP2		WB
Liu et al.[53]	2015	Blast model	cortex	**6h, 12h, 24h, 72h ↑:**<br>S-100β, MBP, ENO1, IL-8, IL-10, iNOS, HIF-1α	**1w ↑:**<br>S-100β, MBP, ENO1, IL-8, IL-10, iNOS, HIF-1α.	ELISA
Liu et al.[54]	2006	CCI	hippocampus, cortex	**2h, 3d ↓:**<br>MBP		WB
McDonald et al.[55]	2018	CCI	NA	**1h, 3h ↑:**<br>LPA		IHC
Mrozek et al.[56]	2019	Weight drop	neocrotex	**4h ↑:**<br>GFAP, UCH-L1 and MMP-9		WB

Ottens et al.[57]	2010	CCI	neocortex	**2d ↑:**<br>P-2, AHCY, AK1, ALB, ALDOC, FETUB, HSP10, HSP70, A1M, NSE, NRGN, PRDX2, PP2A, STIP1, ALDH5A1, SYN2, TF, TKT, SPTN1<br>**2d ↓:**<br>HIBADH, ALDH9A1, ACTA1, ARF1, GPT, ALDOC, AMPH, GOT1, ATP6V1, CBR, COPS2, ENO1, GAPDH, GARS1, PYGB, HSPH1, HSP90, HNRNP, IMPA, IREB1, HSPA4, MAPT, MAP2, NLN, NECAB1/2, NDRG2, PCNP, PDCD6IP, PDHA1, RAB3C, PREP, PPP3CC, SGTA, SOD1, SNAP25, THOP1, TAGLN3, TUBB, UCLH1, SPTN1			WB
Ottens et al.[58]	2008	CCI	cortex	**2d ↓:**<br>MBP			WB
Pabón et al.[59]	2015	CCI	frontal cortex	**3d ↑:**<br>MHCII, Ki-67/nestin			IHC
Rubenstein et al.[60]	2015	CCI	whole brain	**2h, 1d, 2d, 3d ↑:**<br>T-tau, P-tau	**7d, 14d ↑:**<br>T-tau, P-tau	**30d ↑:**<br>T-tau, P-tau	WB/ELISA
Schober et al.[61]	2014	CCI	hippocampus, cortex	**1d, 2d, 3d, ↑:**<br>145 and 120 SBDPs<br>**1d,2d,3d ↓:**<br>SPTN1	**5d, 7d, 14d ↑:**<br>145 and 120 SBDPs<br>**5d,7d,14d ↓:**<br>SPTN1		WB
Thangavelu et al.[36]	2020	PBBI	whole brain	**3d ↑:**<br>BACE1<br>**3d ↓:**<br>APP		**7d ↑:**<br>BACE1, (Aβ)-40;<br>**3d ↓:** APP	qPCR
Yao et al.[62]	2009	PBBI	whole brain	**6-72h ↑:**<br>p43=pro-EMAPII			WB/IHC
Zheng et al.[63]	2020	CCI	hippocampus	**1d ↑:**<br>propionylcarnitine, palmitoylcarnitine, OAT<br>**3d ↑:**<br>CSAD, OAT, BHMT, l-norleucine, 1-Methylhistidine, SLC23A2, propionylcarnitine, palmitoylcarnitine.<br>**1d ↓:**<br>TPH, CHAT, 4-Guanidinobutyric acid, HPRT1, xanthosine.<br>**3d ↓:**<br>adenine and 4-Guanidinobutyric acid			Metabolomics/ bioinformatics

Table S3. Common miRNA targets and differentially expressed proteins.

**Common miRNA targets and proteins (*n* = 57):**	
Foxj1	GLUD1
CXCL1	ALDOA
CCL2	DDAH1
CCL20	MAP2
ACO1	NRGN
C3	PRDX2
GMPS	SYN2
DPYSL2	HIBAH
DPYSL3	ACTA1
DPYSL5	ARF1
WDR1	AMPH
BASP1	COPS2
CFH	GAPDH
ACO2	HSPH1
PLCB1	HSPA4
UBA1	MAPT
STXBP1	NLN
STMN1	NDRG2
SPTBN1	PCNP
ARF3	PDCDP
OXCT1	PDHA1
MDH1	RAB3C
APP	PPP3CC
GDI1	SNAP25
SPTAN1	TAGL3
ANXA11	BACE1
ACSS2	OAT
PGK2	SLC232
PGK1	

Table S4. KEGG pathways identified for miRNAs and proteins with inverse expression relationships and their significance (*p*-value). Pathways highlighted in blue represent common pathways.

**miRNA Pathways:**	***p*-value:**	**Protein Pathways:**	***p*-value:**
MAPK signalling pathway	8.25E-09	Carbon metabolism	4.43E-05
Proteoglycans in cancer	4.80E-08	Biosynthesis of antibiotics	7.85E-05
Prion diseases	7.21E-08	Glycolysis / Gluconeogenesis	0.001523911
GABAergic synapse	7.21E-08	Biosynthesis of amino acids	0.002287116
Endocytosis	3.52E-07	Citrate cycle (TCA cycle)	0.005517089
Pathways in cancer	4.40E-07	Legionellosis	0.016822031
ECM-receptor interaction	1.06E-06	Alzheimer's disease	0.023370569
cAMP signalling pathway	1.98E-06	Metabolic pathways	0.02461046
Thyroid hormone signalling pathway	2.91E-06	Chemokine signalling pathway	0.030417871
Axon guidance	3.00E-06	Chagas disease (American trypanosomiasis)	0.050178535
Arrhythmogenic right ventricular cardiomyopathy (ARVC)	3.64E-06	TNF signalling pathway	0.055511786
Protein processing in endoplasmic reticulum	3.73E-06	Endocytosis	0.062059843
Wnt signalling pathway	3.73E-06	Glyoxylate and dicarboxylate metabolism	0.097126536
Adherens junction	6.10E-06		
Hippo signalling pathway	1.36E-05		
Signalling pathways regulating pluripotency of stem cells	2.23E-05		
Mucin type O-Glycan biosynthesis	2.41E-05		
AMPK signalling pathway	2.46E-05		
FoxO signalling pathway	2.46E-05		
Glycosphingolipid biosynthesis - ganglio series	3.08E-05		
Focal adhesion	3.81E-05		
Regulation of actin cytoskeleton	8.83E-05		
Melanogenesis	0.000127801		

Insulin signalling pathway	0.000133724	
Prostate cancer	0.000149324	
Oestrogen signalling pathway	0.000207177	
Glioma	0.000377612	
Transcriptional misregulation in cancer	0.000427648	
Glutamatergic synapse	0.000433217	
Oxytocin signalling pathway	0.000494775	
Acute myeloid leukaemia	0.000699971	
T cell receptor signalling pathway	0.000699971	
mTOR signalling pathway	0.000699971	
Long-term potentiation	0.000737965	
PI3K-Akt signalling pathway	0.000737965	
Melanoma	0.000833721	
Rap1 signalling pathway	0.000845672	
TGF-beta signalling pathway	0.000881375	
Amphetamine addiction	0.001120869	
Neurotrophin signalling pathway	0.001448782	
Renal cell carcinoma	0.00181256	
Cocaine addiction	0.002407619	
Gap junction	0.002407619	
Osteoclast differentiation	0.002407619	
GnRH signalling pathway	0.002407619	
cGMP-PKG signalling pathway	0.002407619	
Long-term depression	0.003107746	
Basal cell carcinoma	0.003107746	
Colorectal cancer	0.003254237	
Gastric acid secretion	0.003254237	
Chronic myeloid leukaemia	0.003254237	
Morphine addiction	0.004372352	
Endometrial cancer	0.004372352	
Salivary secretion	0.004552374	
Prolactin signalling pathway	0.005279217	
Thyroid hormone synthesis	0.006137191	

Dorso-ventral axis formation	0.007564644	
Type II diabetes mellitus	0.009632621	
ErbB signalling pathway	0.009632621	
HTLV-I infection	0.009730591	
Bacterial invasion of epithelial cells	0.009812095	
Pancreatic cancer	0.011209433	
Choline metabolism in cancer	0.011209433	
Amoebiasis	0.011426703	
Notch signalling pathway	0.012762921	
Platelet activation	0.012762921	
Nicotine addiction	0.012771597	
Adrenergic signalling in cardiomyocytes	0.012777049	
Phosphatidylinositol signalling system	0.012808439	
Cell adhesion molecules (CAMs)	0.012808439	
N-Glycan biosynthesis	0.016223308	
Hepatitis B	0.016223308	
Ubiquitin mediated proteolysis	0.016223308	
Vasopressin-regulated water reabsorption	0.017235861	
Hedgehog signalling pathway	0.017235861	
Adipocytokine signalling pathway	0.018997795	
Inositol phosphate metabolism	0.019254734	
Calcium signalling pathway	0.019740063	
Small cell lung cancer	0.022329205	
TNF signalling pathway	0.022329205	
Ras signalling pathway	0.023787094	
Endocrine and other factor-regulated calcium reabsorption	0.034328134	
Lysine degradation	0.034328134	
mRNA surveillance pathway	0.034328134	
Circadian rhythm	0.035635515	
Progesterone-mediated oocyte maturation	0.044951329	
Central carbon metabolism in cancer	0.044994013	
